# A33 EOSINOPHILS PROMOTE *SALMONELLA* TYPHIMURIUM COLONIZATION IN THE MURINE SMALL INTESTINE

**DOI:** 10.1093/jcag/gwad061.033

**Published:** 2024-02-14

**Authors:** R D FitzPatrick, J R Noone, R A Cartwright, D M Gatti, T Brosschot, J M Lane, M Zafarullah, I Kroker Kimber, L Reynolds

**Affiliations:** . University of Victoria, Victoria, BC, Canada; . University of Victoria, Victoria, BC, Canada; . University of Victoria, Victoria, BC, Canada; . University of Victoria, Victoria, BC, Canada; . University of Victoria, Victoria, BC, Canada; . Biochemistry & Microbiology, University of Victoria, Victoria, BC, Canada; . University of Victoria, Victoria, BC, Canada; . University of Victoria, Victoria, BC, Canada; . University of Victoria, Victoria, BC, Canada

## Abstract

**Background:**

During inflammatory bowel disease (IBD) both the number of intestinal eosinophils and susceptibility to enteric bacterial infections can drastically increase. It is unknown, however, whether eosinophils recruited to the tissues lining the gastrointestinal (GI) tract during severe disease help to control pathogenic bacterial colonization or whether their presence promotes bacterial infection. No studies to date have examined the role of eosinophils during an acute bacterial infection in the small intestine.

**Aims:**

Our research aims to uncover the role of eosinophils during an enteric *Salmonella* infection in mice and we hypothesize that eosinophils undergo phenotypic changes in the presence of *Salmonella* and aid in the clearance of this enteric bacterial pathogen.

**Methods:**

To assess how the presence of *Salmonella* impacts the frequency and phenotype of eosinophils we performed flow cytometry on eosinophils isolated from mice 3-days post-oral infection with *Salmonella enterica* serovar Typhimurium and compared these findings to naïve BALB/c controls. To determine if eosinophils are essential for controlling *Salmonella* infection we quantified levels of *Salmonella* colonization along the GI tract, liver and spleen of wild-type and eosinophil-deficient (ΔdblGATA) littermate mice by plating homogenized tissues on LB agar.

**Results:**

Following an oral *Salmonella* infection we found higher frequencies of eosinophils in the gut-draining mesenteric lymph nodes compared to uninfected mice. Intestinal lamina propria-resident eosinophils in the small intestine and colon displayed altered phenotypes following infection in an intestinal-region specific manner: during infection, in the small intestine eosinophils increased their expression of MHCII, and in the colon eosinophils displayed elevated granularity. When wild-type and ΔdblGATA mice were pre-treated with the antibiotic streptomycin 1-day prior to infection to confer robust *Salmonella* burdens in the small intestine we found a significant reduction in *Salmonella* colonization in the ileum of the small intestine in ΔdblGATA mice compared to their wild-type littermates. These data suggest eosinophils have a role in *promoting* colonization by this enteric bacterial pathogen.

**Conclusions:**

Our data contribute to the growing evidence that microbes can influence the phenotype of intestinal eosinophils and demonstrate that the influence of and requirements for eosinophils during bacterial infection is highly context-dependent. We found that eosinophils are not essential for controlling an acute enteric *Salmonella* infection and instead they promote *Salmonella* colonization in the small intestine.

**Funding Agencies:**

CIHRTRIANGLE

